# Inverse Lithography Technology (ILT) Under Chip Manufacture Context

**DOI:** 10.3390/mi17010117

**Published:** 2026-01-16

**Authors:** Xiaodong Meng, Cai Chen, Jie Ni

**Affiliations:** 1 School of Integrated Circuits, Tsinghua University, Beijing 100084, China; 2 Advanced Manufacturing EDA Co., Ltd., Shanghai 201204, China

**Keywords:** inverse lithography technology, computational lithography, process window, pattern fidelity

## Abstract

As semiconductor process nodes shrink to 3 nm and beyond, traditional optical proximity correction (OPC) and resolution enhancement technologies (RETs) can no longer meet the high patterning precision needs of advanced chip manufacturing due to the sub-wavelength lithography limits. Inverse lithography technology (ILT), a key part of computational lithography, has become a critical solution for these issues. From an EDA industry perspective, this review provides an original and systematic summary of ILT’s development and applications, which helps integrate the scattered research into a clear framework for both academic and industrial use. Compared with traditional OPC, the latest ILT has three main advantages: (1) better patterning accuracy, as a result of the precise optical models that fix complex optical issues (like diffraction and interference) in advanced lithography systems; (2) a wider process window, as it optimizes mask designs by working backwards from the target wafer patterns, making lithography more stable against process changes; and (3) stronger adaptability to new lithography scenarios, such as High-NA EUV and extended DUV nodes. This review first explains ILT’s working principles (the basic concepts, mathematical formulae, and main methods like level-set and pixelated approaches) and its development history, highlighting key events that boosted its progress. It then analyzes ILT’s current application status in the industry (such as hotspot fixing, full-chip trials, and EUV-era use) and its main bottlenecks: a high computational complexity leading to long runtime, difficulties in mask manufacturing, challenges in model calibration, and a conservative market that slows large-scale adoption. Finally, it discusses promising future directions, including hybrid ILT-OPC-SMO strategies, improving model accuracy, AI/ML-driven design, GPU acceleration, multi-beam mask writer improvements, and open-source data to solve data shortage problems. By combining the latest research and industry practices, this review fills the gap of comprehensive ILT summaries that cover the principles, progress, applications, and prospects. It helps readers fully understand ILT’s technical landscape and offers practical insights for solving the key challenges, thus promoting ILT’s industrial use in advanced chip manufacturing.

## 1. Introduction

**Figure 1:**
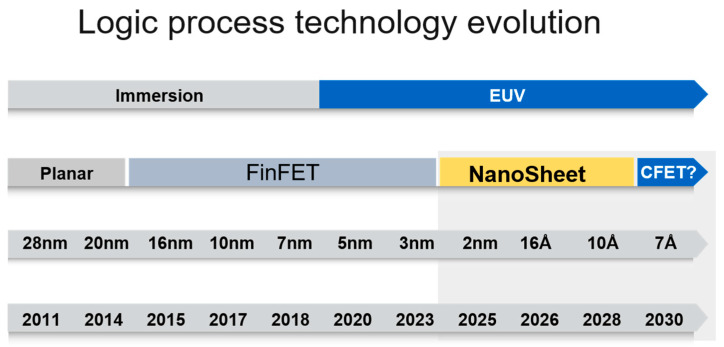
Logic process technology evolution. Process nodes continue to shrink year by year—entering the 2 nm era in 2025 [7].

Lithography is the core technology in IC manufacturing, playing a crucial role in determining the performance, cost, and miniaturization of ICs [1,2,3,4,5,6]. It is used to transfer the circuit pattern on the mask to the semiconductor substrate, enabling the precise fabrication of various components in the IC. The continuous development of lithography technology has been a major driving force behind the miniaturization and performance improvement of ICs. As shown in Figure 1, according to the YOLE Intelligence [7] report, as the feature size of ICs continues to shrink, the requirements for lithography technology become more stringent. When the minimum line width in the device is smaller than the exposure wavelength of the lithography machine, the diffraction and interference effects of the adjacent patterns can cause a distortion of the image on the wafer, known as the optical proximity effect (OPE). The practice of correcting the pattern on the mask appropriately to obtain the same pattern as the design on the wafer is called optical proximity correction (OPC).

**Figure 2:**
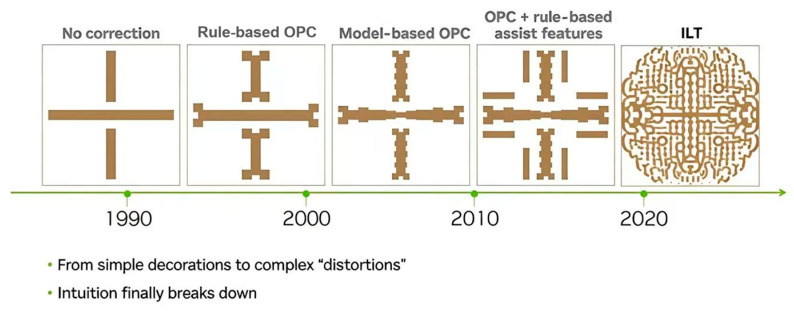
Demand for mask correction varies in different eras due to technological advancements and increasing need [3]. The milestones marked on the timeline indicate when each correction method became a prominent approach, not its initial inception. For example, ILT gained significant attention after 2020.

As illustrated in Figure 2 [3], the requirements for mask correction have evolved across different technological eras, driven by advancements in semiconductor manufacturing and increasingly stringent performance demands. Overall, OPC technology has undergone a progressive refinement, transitioning roughly through four distinct generations: rule-based OPC (RB-OPC), model-based OPC (MB-OPC), rule/model-based sub-resolution assist feature (SRAF) insertion, and model-driven pixel-level optimization employed in inverse lithography technology (ILT). This evolution has been accompanied by a corresponding increase in algorithmic and computational complexity.

The earliest iteration, RB-OPC, operated on predefined correction heuristics applied globally to mask layouts. These rules typically established bias tables based on critical geometric attributes such as line width and pitch, enabling the automated modification of qualifying patterns through edge-displacement operations—a technique known as edge-based correction. A significant advancement came with the integration of SRAF into RB-OPC frameworks: by inserting sub-resolution auxiliary features (typically linear or block-shaped structures), the process window for semi-isolated and isolated patterns was substantially expanded. Despite their simplicity, RB-OPC and rule-based SRAF methodologies remained effective solutions for mitigating OPE.

As IC technology advanced to finer process nodes, the shrinking critical dimensions (CDs) and increasingly complex pattern topologies necessitated more sophisticated correction approaches, leading to the development of MB-OPC. This paradigm shift introduced lithographic imaging models—incorporating both optical and photoresist response physics—thereby framing OPC as a mathematical optimization problem for mask patterning. Leveraging computational optimization algorithms, MB-OPC calculates optimal mask geometries by minimizing the edge placement error (EPE), defined as the Euclidean distance between the model-predicted contours and target layouts at the pre-specified evaluation points. The direct correlation between the minimized EPE and improved pattern fidelity makes it the primary cost function in modern model-based correction workflows [8].

ILT has emerged in response to the challenges faced by traditional lithography techniques as the feature size of ICs continues to shrink [9]. When the feature size of ICs is reduced to 45 nm or less, the complexity of OPE greatly increases. The correction strategy based on line segment displacement will be limited by the topology and inherent layout of the original layout, and its potential solution space will become limited, making it difficult to obtain ideal correction results [10]. ILT begins by discretizing the mask into pixel arrays, obviating the need for the manual segmentation of polygons’ edges in mask. Subsequently, via iterative mathematical computations, the pixelated mask undergoes evolution along the gradient descent direction of the lithographic error function, continuing until the function reaches convergence. By breaking free from the topological constraints inherent in the original design layout, ILT exhibits greater flexibility compared to the edge-segmentation-based correction strategies of conventional OPC. This enhanced flexibility enables ILT to achieve superior imaging fidelity and a more expansive process window in advanced lithographic applications [5].

## 2. The Working Principle of ILT

### 2.1. The Basic Concepts and Processes

Lithography is a key procedure in the manufacturing process of ICs, and its resolution and accuracy significantly impact the performance of the microelectronic devices. With the development of semiconductor technology and the continuous improvement in technology nodes, especially when the technology nodes are less than 90 nm [11], the impact of the OPE on the lithography process is becoming increasingly evident. Therefore, it has become a key issue to narrow the gap between the lithography process control capability and the lithography process window and enhance the efficiency and performance of the lithography process.

Currently, for mitigating the impact on OPE, MB-OPC has become a standard practice [12]. In MB-OPC, the mask is first segmented into a series of movable line segments according to pre-defined rules. Lithography simulations are then performed on the mask, and the line segments are iteratively adjusted based on the simulation results to correct pattern distortions. However, as the feature size of ICs shrinks to 45 nm and below, the complexity of OPE increases significantly. Traditional correction strategies based on line segment displacement face limitations imposed by the original layout topology and fixed design constraints, leading to a constrained solution space that struggles to achieve the optimal correction results.

As a novel resolution enhancement technique (RET), ILT can achieve a higher imaging quality and a larger process window [13]. The strategy of ILT is to pixelate the layout, and then optimize the position and size of each pixel to obtain the best results. Thus, ILT is known as pixel-based OPC (PB-OPC). The advantage of this method is that it does not have the limitation based on the fixed line segment displacement in traditional OPC. By directly processing pixels instead of moving line segments, ILT provides greater flexibility in layout modification, enabling it to cope with more complex OPE [14].

**Figure 3:**
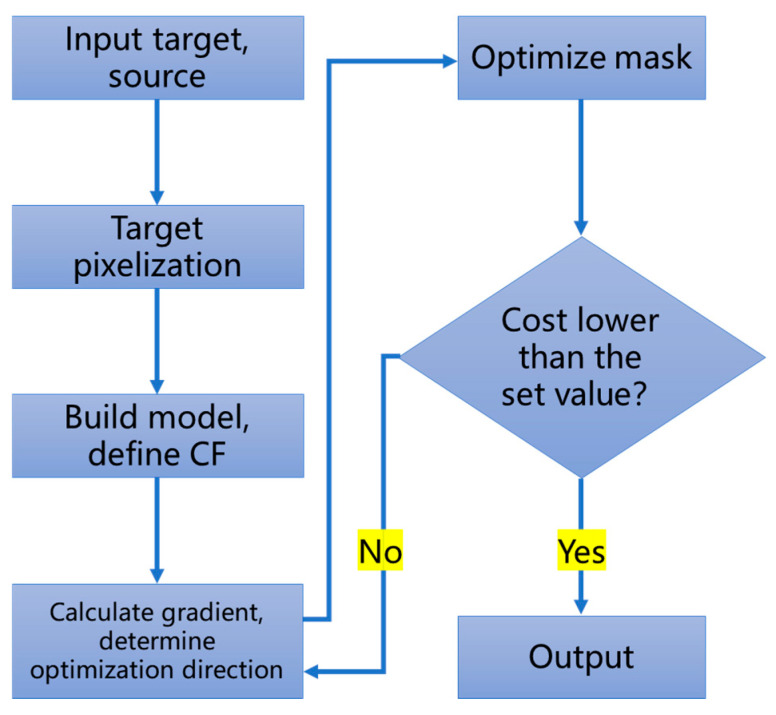
The general process of ILT. Formulation of a cost function as the discrepancy between the printed wafer pattern and the target layout within the imaging region, with optimization direction determined by the nature of the cost function—e.g., gradient-based descent for differentiable (continuous) cost functions [15].

The workflow of mask pattern generation using ILT is illustrated in Figure 3. The process commences with defining inputs such as the target pattern, illumination source parameters, and initial mask configuration, followed by constructing a pixelated mask model. A lithography model is then established to simulate the aerial image formation, where the sigmoid function is typically employed to characterize the photoresist threshold behavior. This allows the prediction of the latent image profile on the photoresist.

Subsequently, a cost function (CF) is formulated to quantify the discrepancy between the simulated wafer image and the target pattern. The choice of optimization algorithm hinges on the nature of the CF: For continuously differentiable formulations, gradient-based methods are commonly adopted to navigate the solution space. Advanced variants such as steepest descent, momentum-based optimization, adaptive gradient, and adaptive moment estimation offer distinct strategies to enhance convergence speed and stability. The method of iteration is used to correct a mask according to the set step length after calculating the optimization direction for each iteration until the satisfactory mask is found. The CF can include many additional elements, such as the mask manufacturing error and its impact on wafer printing [15]. For example, in level-set-based inverse lithography, the inverse lithography problem is addressed as an obstacle reconstruction problem or an extended nonlinear image restoration problem, and then solved by a level-set time-dependent model with finite difference schemes [16].

### 2.2. Mathematical Formulation and Optimization Algorithms

Mathematical description and algorithms are at the heart of ILT. The imaging model of optical lithography is the foundation for developing inverse lithography algorithms. It describes the relationship between the mask pattern, illumination source, and the resulting image on the resist. When ILT is used to calculate the mask, the pattern required by the target layout, a pair of mathematical expressions of the inverse problem are usually used to describe this process [10]:

(1) $$Contour=Litho\left(mask\right)$$

(2) $${mask}^{*}={Litho}^{-1}(z)$$

where Litho() represents a forward lithography calculation function, which can be calculated using a lithography simulation system. Mask is the mask pattern, and contour is the wafer image generated by the lithography simulation. Litho^−1^() represents its inverse function, z is the original design layout, i.e., the target layout, and mask* is the corresponding mask when the contour of lithography result is z. If the expression of Litho^−1^() can be obtained, the solution mask* for inverse lithography can be directly calculated.

During the inverse lithography process, the CF serves as an optimization criterion, guiding iterative mask modifications to minimize the discrepancies between the simulated and target patterns. The basic form of a CF in ILT is shown below [17]:

(3) $$CF\left[mask\right]=\iint {w_{p}*\left(Actual-goal\right)}^{2}dxdy$$

And this CF can be extended to capture many process conditions, for example,

(4) $$CF\left[mask\right]=\sum_{pw}\iint {w_{p}*\left(Actual-goal\right)}^{2}dxdy$$

in this context, (actual—goal) mathematically defines the spatial error metric between the simulated pattern and the target specification. The topological weight W_p_ allows for attribute-specific weighting, where critical features can be assigned higher weights to prioritize their accuracy, and pw represents the multiple process window conditions.

ILT employs gradient-based optimization by computing ∂Cost/∂Mask to derive the optimal global mask modifications that minimize the lithographic CF. The mask undergoes iterative updates according to the equation below, where the gradient Gm is scaled by a damping factor to ensure numerical stability and convergence.

(5) $${Mask}_{M+1}={Mask}_{M}-G_{m}(x,y)\delta$$

It should be noted that this overview represents a highly simplified approximation of ILT; for a more rigorous and comprehensive mathematical formulation, readers are referred to the extensive body of literature on this subject, e.g., [18,19,20,21]. In terms of algorithm development, ILT was first proposed by B. E. A. Saleh and S. I. Sayegh [22]. A few years later, Saleh and Nashold [23] described an algorithm using a sequence of projection operators to find a band-limited function, corresponding to a continuous-tone or gray-scale mask that optimized the desired image. At the beginning of the development of ILT, Pati et al. [24] proposed the projection onto the convex sets (POCS) algorithm, which is rooted in convex set optimization theory, to mitigate the image distortion induced by binary masks and phase-shift masks. Meanwhile, Sherif et al. [25] proposed a binary mask optimization method based on the incoherent diffraction lithography system, which expresses the ILT as mixed-integer linear programming and solves it through the branch-and-bound method. Furthermore, random-pixel programming [26], genetic algorithm [27,28], and nonlinear programming [29] discrete algorithms have been proposed one after another to solve ILT-related problems.

However, discrete methods have inherent limitations: due to the fact that discrete optimization only optimizes in discrete space, the entire optimization process may not converge stably, and there may be problems with a slow convergence speed [10]. Amyn Poonawala et al. [30] first converted this discrete optimization problem into a constrained continuous one, and further transformed it into an unconstrained continuous optimization problem by introducing intermediate variables. Once unconstrained, it can be solved using various continuous optimization algorithms like gradient descent. Recently, Ma et al. [31] proposed a better solution for multi-patterned mask design by simultaneously optimizing masks and decomposing layouts. Yu et al. [32] introduced the Adam search algorithm into gradient-based methods. Compared to the commonly used stochastic gradient descent (SGD), this algorithm adjusts the learning rate during random search by calculating the first and second moments of the gradient, achieving faster convergence.

New algorithms have been proposed to improve the computational efficiency and imaging performance of ILT. For example, a fast inverse lithography based on the dual-channel model-driven deep-learning (DMDL) method has been developed [33]. The architecture of the DMDL network is derived from the inverse optimization model under a gradient-based ILT framework. A dual-channel structure is introduced to simultaneously modify the mask contour and insert SRAFs, improving the lithography image fidelity. An unsupervised training strategy based on the auto-decoder is developed to avoid the time-consuming labelling process. The superiority of DMDL over the state-of-the-art ILT method has been verified in both the computational efficiency and image fidelity obtained on the semiconductor wafer [33]. In the area of multi-objective optimization, the multi-objective ILT and the hybrid dynamic priority (HDP) algorithm have been investigated and developed [9]. High-performance images with a high fidelity and high uniformity have been obtained at multi-field and multi-clip areas across the die. The HDP algorithm can improve the uniformity of images at full-field points by up to 31.1% compared to the current multi-objective optimization algorithms in the case of multi-field wavefront error-aware source mask optimization (SMO) [9]. Furthermore, in applications of ILT to various lithography systems—such as extreme ultraviolet lithography (EUVL)—researchers have successfully printed test patterns despite the large spherical aberrations in the single-spherical-mirror projection optics by designing masks with an adjoint-based gradient descent optimization algorithm [34].

### 2.3. Mainstream Implementation Methods

While programming approaches and optimization algorithms for ILT can vary widely, the mainstream implementation methods that have been practically realized and widely adopted in the industry can be broadly categorized into four primary types.

#### 2.3.1. Level-Set Method

One important method about ILT is the level-set method, which was proposed by Stan Osher and James Sethian [18] in 1988. Luminescent (Luminescent Technologies, Inc. (Santa Clara, CA, USA)) first applies the level-set method to ILT to solve the ILT runtime problem [19,35,36,37,38]. The goal of the level-set method is to minimize CF by reducing the number of variables and solving topological discontinuities. The main idea of the level-set method is to convert a 2D closed curve into a 3D surface, treating the 2D closed curve as a set of intersection lines between the 3D surface and the zero plane; hence, it is called a level set. Building on this principle, starting in 2003, Luminescent developed the ILT approach based on the level-set method, aiming to enhance mask optimization efficiency, reduce complexity, and thereby shorten runtimes [13].

**Figure 4:**
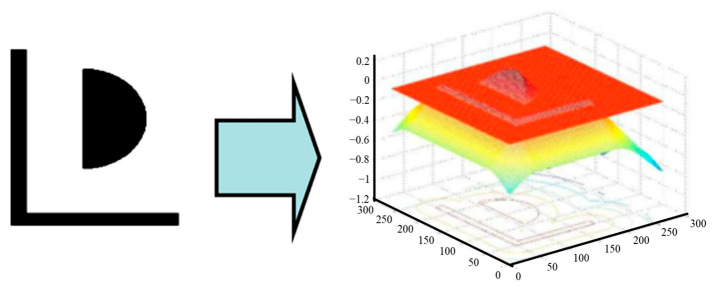
The process of converting a 2D closed curve into a 3D surface, embedding a 2D closed contour as the zero level set of a 3D signed distance function in ILT [13]. Different colors in the right panel represent different level sets of the signed distance function.

As shown in Figure 4, a distance function can be defined such that, for any point (x, y), its value (z) corresponds to the shortest distance from that point to the nearest edge of the 2D curve. The surface represented by this function intersects the xy-plane in a 2D shape, and this shape at the zero level set (on the xy-plane) is exactly the original 2D curve [13].

The level-set mathematical method solves the problem of 2D curve evolution. When designing the ILT algorithm, the lithography model is used to determine the optimization direction and velocity of each point on the mask, and the level set is used to achieve the evolution of the mask [39].

**Figure 5:**
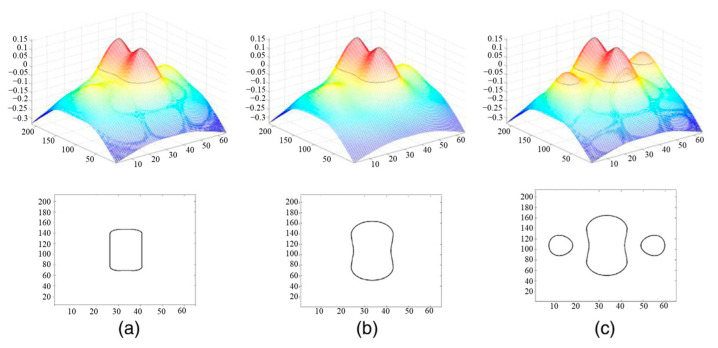
The schematic diagram of generating SRAF by level-set method [13]. Different colors represent different level sets of the signed distance function. (**a**–**c**) illustrate the key steps in generating SRAFs. The SRAFs are formed between step (**b**) and step (**c**) by elevating the surface around the main contact hole. As this elevated surface intersects the zero-level plane (i.e., the xy-plane), the SRAFs emerge.

As shown in Figure 5, a main pattern (a contact hole, in this case) on the mask exhibits 2 SRAFs by raising the surface around the main contact hole during the optimization process, which undergoes a sudden change in the evolution of the 2D curve from one closed curve to three closed curves, while the constructed 3D surface remains a continuous surface. When the surface passes through the plane corresponding to a level of zero (the xy-plane), the SRAFs appear. Construct a 3D surface using the level-set method, and transform the lithography pattern composed of closed curves into the intersection line between the surface and the zero-value plane. The emergence of SRAF makes 2D functions discontinuous. By using the level-set method to transform them into 3D surfaces that can be continuous, ILT optimization can be expressed as a general multivariate optimization problem and solved using standard optimization algorithms such as the conjugate gradient method.

#### 2.3.2. Intel Pixelated ILT

Another ILT method is the Intel pixelated ILT, which was introduced in 2007 and represents a significant advancement in lithography optimization [40]. This approach utilizes alternating phase-shift masks (Alt-PSMs) to enhance the resolution and employs a pixel-based design to satisfy mask rules and reduce computational complexity. Unlike previous methods that focused on pattern edges, Intel’s method simplifies the problem by mapping the design onto large pixels with only two degrees of freedom (0° and 180° phase shifts). This reduction in complexity allows for more efficient computation while maintaining pattern fidelity and symmetry.

**Figure 6:**
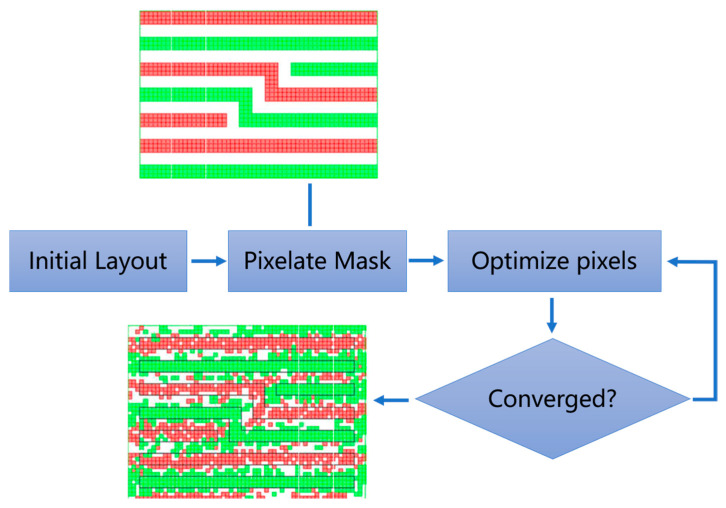
Pixel optimization flow. A phase-coloring algorithm is applied to assign phases to polygons to maximize patterning fidelity, followed by pixel-level optimization to resolve phase conflicts introduced by the coloring process [40].

The specific operation steps are shown in the following figure (Figure 6). The process begins with importing an initial layout, which comprises polygons on a target layer. Subsequently, a phase-coloring algorithm is applied to these polygons, with the aim of maximizing the patterning accuracy. It should be noted that phase conflicts may arise from the phase-coloring algorithm; here, pixel optimization is employed to address such conflicts. Following this, the polygons are converted into a pixelated form based on a fixed pixel grid. These pixels then undergo an iterative optimization process, ultimately leading to a converged pixel layout, as illustrated in Figure 6 [40].

Despite its advantages, the Intel method faced challenges, particularly with pixel size limitations. The 100 nm pixel size on the mask, while smaller than the 193 nm wavelength, introduced strong 3D mask effects, especially for Alt-PSMs. To address this, Intel developed a rigorous 3D mask model. Additionally, the method required significant computational resources, necessitating innovations like GPU acceleration and stitching techniques to manage full-chip designs. Intel also demonstrated the practicality of its approach by successfully taping out an experimental mask for a 65 nm node microprocessor, achieving yields comparable to conventional production methods.

The Intel pixelated ILT method laid the groundwork for future advancements, including the integration of multibeam mask writers and GPU-accelerated computing. These innovations not only addressed the runtime and mask writing challenges but also paved the way for the adoption of curvilinear ILT solutions in production environments. The method’s success in improving process windows and reducing defects underscored its potential for addressing the complexities of advanced semiconductor manufacturing.

#### 2.3.3. Frequency-Domain Curvilinear ILT

Another ILT method is about calculating curvilinear ILT in the frequency domain. The Gauda/D2S (Gauda, Inc. (San Jose, CA, USA), which was acquired by D2S, Inc., in 2014) [41] approach to ILT represents a significant advancement in the field, leveraging GPU acceleration and a band-limited frequency-domain method to overcome the traditional challenges in ILT implementation. Unlike the Luminescent level-set method and the Intel pixelated approach, which operate in the spatial domain, Gauda/D2S introduced a novel optimization framework that solves the ILT problem in the frequency domain. The method involves transforming the mask from the spatial domain to the frequency domain, performing lithography model calculations in the frequency domain, and using the closed-form integral modeling provided by the Hopkins formula to transform the equation into a four-dimensional convolution between the optical system transfer function and the mask function M. The time-domain convolution is equivalent to frequency-domain multiplication, and the convolution operation is transformed into Fourier-transform and multiplication operations that are more suitable for parallel operations.

**Figure 7:**
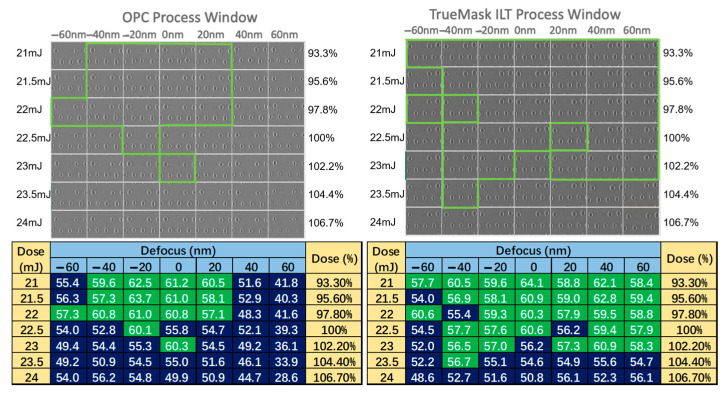
Process window CD measurements. Top row: Process windows for (**left**) conventional OPC and (**right**) TrueMask ILT. Bottom row: Corresponding SEM images at various focus and exposure dose conditions under the respective processes. The highlighted regions are within an acceptable process window [42].

The Gauda/D2S method also integrates mask-rule checking (MRC) to ensure that the final ILT masks meet manufacturing requirements, correcting any features that violate the minimum dimensions [42]. By maintaining symmetry and avoiding small features, the approach not only improves the lithographic results but also enhances mask manufacturability. As shown in Figure 7, the system demonstrated exceptional performance, producing full-chip ILT solutions within a day or two, with process-window improvements exceeding 100% [42]. This development marks a pivotal moment in the history of ILT, transitioning it from an academic concept to a practical production reality.

#### 2.3.4. Machine-Learning–Assisted ILT

The three methods described above are generally hindered by a high computational complexity, limiting their widespread adoption in semiconductor manufacturing. In recent decades, the integration of machine learning with ILT has emerged as a promising direction to alleviate this bottleneck. By leveraging data-driven models, ML-enhanced ILT aims to significantly improve computational efficiency while maintaining or even enhancing patterning fidelity—making it a key trend for scaling ILT to advanced technology nodes.

Conventional ILT methods, while physically rigorous, suffer from prohibitive computational costs due to iterative optimization in high-dimensional mask spaces, limiting their scalability in advanced-node semiconductor manufacturing. Machine learning circumvents this bottleneck by learning the complex, nonlinear mapping from target wafer patterns to optimal mask representations through data-driven inference. Unlike traditional approaches that rely on handcrafted features and explicit physical solvers, deep learning leverages multi-layer neural architectures to autonomously extract hierarchical features and enable end-to-end optimization, thereby capturing intricate optical and resist effects without explicit modeling. Moreover, deep learning excels in handling large-scale, high-dimensional lithographic datasets, making it especially well-suited for high-precision mask synthesis, process window enhancement, and robustness against focus and dose variations. Consequently, integrating deep learning into ILT not only accelerates mask optimization by orders of magnitude but also maintains or even improves pattern fidelity, positioning it as a critical enabler for next-generation computational lithography [43].

Early efforts to integrate machine learning into ILT date back to Jia et al. [44], who first framed mask design as a learning task and incorporated focus variation as a stochastic variable to enhance robustness. Subsequently, Luo et al. explored the use of a multi-layer perceptron for mask pattern prediction [45] and later proposed an SVM-based layout retargeting method to generate high-quality initial masks, thereby accelerating ILT convergence [46]. Kumar et al. introduced a clustering-based hybrid OPC approach that reduces the simulation time compared to conventional workflows [47]. Despite these advances, traditional machine-learning and deep-learning methods face inherent limitations—such as complex architectures, limited physical interpretability, and unpredictable generalization—prompting the emergence of model-driven deep learning. This paradigm improves the prediction reliability by embedding prior physical knowledge, such as lithographic imaging models, into the neural network architecture [33,48,49,50]. For instance, model-constrained convolutional neural networks (MCNNs) are constructed by unfolding and truncating iterative ILT solvers, leveraging physics-based decoders for unsupervised training [15,50]. Similarly, Jiang et al. developed a fast, learning-based mask printability prediction framework that significantly speeds up OPC while maintaining or improving patterning fidelity [51]. Compared to conventional machine learning, deep learning offers superior capabilities in automated feature extraction, large-scale data processing, and modeling complex nonlinear lithographic responses, making it particularly promising for next-generation ILT applications.

**Figure 8:**
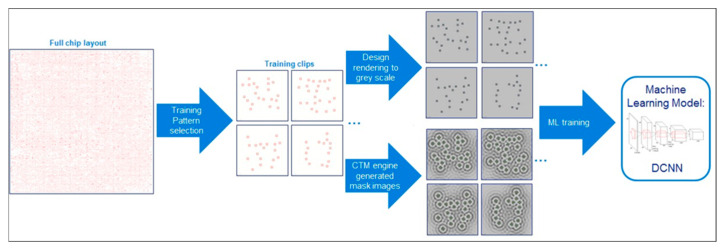
The training flow of our proposed machine-learning SRAF method [13].

Building upon the paradigm of model-driven ML, recent studies have demonstrated practical implementations of DL in ILT. Notably, ASML Brion [52] demonstrated a free-form ILT engine that uses a CNN to train an ILTDL model, as shown in Figure 8. In this framework, the trained DL-ILT model first generates an initial SRAF layout, which is then refined and used as the starting point for the conventional ILT optimizer. Experimental results show that this DL-based initialization significantly reduces the overall computation time for SRAF generation without compromising pattern fidelity [13]. Complementing this effort, Shi et al. [53] proposed a CNN-based approach for the automatic design of optimal feature vectors to minimize errors during lithographic feature extraction. Ye et al. [54] put forward a lithography model grounded in generative adversarial networks (GANs), enabling the rapid prediction of lithography contours from masks. Kumar et al. [55] adopted a multi-stage regression method to enhance the accuracy of lithography models. More recently, Zhong et al. [56] proposed a CNN-based model for layout decomposition and quality prediction, which was incorporated into an efficient collaborative optimization framework integrating layout decomposition and inverse lithography.

Moreover, recent work has further expanded the scope of deep learning in ILT through generative modeling and full-chip optimization. Zhang et al. [57] employed a variational autoencoder (VAE) for mask design, improving the model’s generalization ability and stability through regularization terms and Bayesian inference. Zhao et al. [58] proposed a CNN network based on self-calibrated convolution optimization to improve the computational efficiency of ILT, where each position adaptively encodes long-range contextual information. Yang et al. [59] developed a GAN model with an OPC-oriented flow, ILT pre-training, and enhanced generator to improve mask optimization, reducing OPC steps and boosting printability. Chen et al. [60] introduced deep agile mask optimization (DAMO), a deep-learning-enabled full-chip OPC system with a deep lithography simulator (DLS), a deep mask generator (DMG), and a custom layout splitting algorithm, demonstrating superior performance over both academic and industrial state-of-the-art solutions.

### 2.4. Comparison and Analysis of Mainstream Methods

Four mainstream ILT implementation methods—the Level-Set Method, Pixelated ILT, Frequency-Domain Curvilinear ILT, and ML-Assisted ILT—exhibit distinct characteristics in computational efficiency, mask manufacturability, and application scope. Table 1 presents a concise comparison of their core performance, while subsequent sections provide an in-depth analysis of their technical trade-offs and practical deployment considerations.

The Level-Set Method strikes a balance between performance and practicality. By converting 2D mask contour optimization into 3D surface evolution, it reduces the variable dimensionality compared to pixelated approaches, achieving a stable convergence. Its generated smooth curvilinear masks require no additional post-processing, making it compatible with mainstream multi-beam mask writers. However, its computational efficiency declines significantly for full-chip-scale patterns, limiting its application to medium-small critical structures like logic gate hotspots and SRAM cells, especially in DUV extension node R&D.

Pixelated ILT (represented by Intel’s solution) prioritizes patterning precision at the cost of efficiency and manufacturability. Its pixel-wise optimization mode enables ultra-high pattern fidelity, meeting the strict EPE requirements for advanced logic nodes. Nevertheless, the massive variable dimensionality leads to an extremely long runtime, which can only be mitigated by multi-CPU/GPU clusters. Additionally, its output often features jagged edges and irregular pixel clusters, necessitating post-processing steps like edge smoothing to ensure manufacturability. This method is thus only feasible for leading foundries (e.g., TSMC and Intel) with abundant computing resources.

Frequency-Domain Curvilinear ILT excels in efficiency for regular patterns. By leveraging Fourier transform to shift optimization from the spatial to the frequency domain, it effectively filters high-frequency components that cause mask complexity, achieving fast convergence for large-area periodic structures such as DRAM/NAND memory cell arrays. Its generated regular, smooth masks boast excellent manufacturability, making it the preferred choice for memory chip mass production where efficiency and yield are critical. However, its performance degrades for irregular logic patterns, limiting its scope to scenarios with repetitive structures.

ML-Assisted ILT breaks the traditional trade-off between efficiency and precision through data-driven optimization. While the training stage requires large-scale high-quality layout data and is time-consuming, the inference stage can complete mask optimization 10–100× faster than traditional methods. By incorporating manufacturability rules into training labels, it can control the mask complexity within acceptable limits. This method is well-suited for mass production scenarios like hotspot repair for mature and advanced nodes, as well as semi-repetitive patterns such as logic standard cells. Its main limitation lies in its heavy reliance on data quality and quantity, which restricts its application in scenarios lacking sufficient training data.

In summary, the selection of ILT methods depends on core application demands: full-chip optimization for advanced logic nodes prioritizes Pixelated or ML-Assisted ILT (with hardware acceleration); memory chip mass production favors Frequency-Domain Curvilinear ILT for efficiency and manufacturability; while Level-Set Method is ideal for medium–small-scale critical pattern optimization in R&D stages.

## 3. Historical Evolution and Research Progress

### 3.1. Evolution of Lithography Techniques

Lithography accounts for 30% to 40% of the manufacturing cost of ICs, and is the most complex, expensive, and critical step in the IC manufacturing process [10]. The semiconductor industry has witnessed a continuous evolution of lithography techniques over the years. Early lithography techniques, such as optical lithography, were based on the use of visible or ultraviolet light to transfer patterns onto the semiconductor substrate. However, as the demand for smaller feature sizes increased, new techniques were developed.

EUVL emerged as a promising technology for the next-generation semiconductor manufacturing. EUVL uses extreme ultraviolet light with a wavelength of 13.5 nm, enabling the fabrication of features with sub-10 nm half-pitches. This technology has the potential to extend Moore’s law and meet the requirements of advanced IC manufacturing [61]. Electron beam lithography (EBL) is another important technique that offers high-resolution patterning. EBL uses a focused electron beam to write patterns directly on the resist, achieving resolutions down to a few nanometers. However, EBL suffers from a low throughput, making it more suitable for prototyping and small-scale production [62] Nanoimprint lithography (NIL) has also gained significant attention. NIL is a cost-effective and high-throughput method for replicating nanostructures. It involves imprinting a pattern from a mold onto a resist, which can be used for various applications, including the fabrication of semiconductor devices. For example, jet and flash imprint lithography (J-FIL) has been transitioned from research to a commercial fabrication infrastructure for leading-edge semiconductor ICs [61]. In addition, other techniques such as X-ray lithography and ion beam lithography have also been explored. X-ray lithography uses synchrotron radiation to achieve deep, high-resolution features, while ion beam lithography can be used for high-precision maskless patterning [6].

The cost of EUV lithography machines is very high, so they are currently only deployed at a small number of semiconductor manufacturers. Moreover, due to trade restrictions imposed by the United States on China, there are currently no EUV lithography machines deployed domestically. Currently, mainstream lithography systems still use 193 nm wavelength light sources.

### 3.2. Historical Trends and Resurgence of ILT Research

ILT was first proposed by B. E. A. Saleh and colleagues at the University of Wisconsin-Madison [13], in 1981. Saleh and Sayegh [22] developed an optimized photomask using a simulated annealing variant with pixel flipping, while Saleh and Nashold [23] later (several years after 1981) proposed a projection operator-based algorithm to find a band-limited function for continuous-tone or gray-scale mask optimization. Since 2000, particularly as process nodes entered the 90 nm stage, OPC technology began to be widely adopted, and ILT also entered a period of rapid development. Although it experienced periodic plateaus during this time, the overall trend has remained progressive. This trend is evident in the statistics of annual ILT-related article publications compiled in this study.

**Figure 9:**
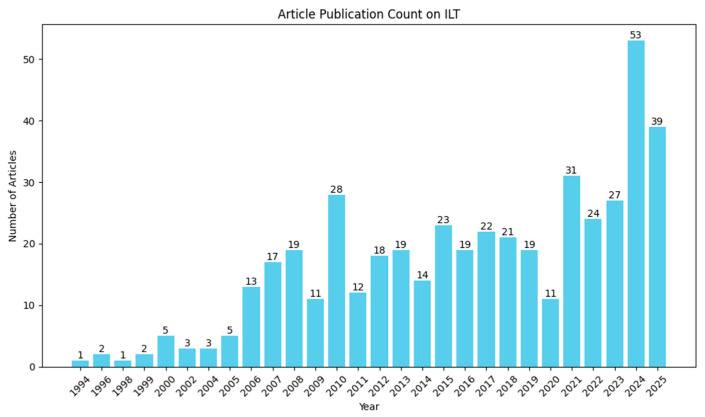
Annual number of publications on the topic of “inverse lithography technology”.

Figure 9 presents the annual number of publications on the topic of “inverse lithography technology” retrieved by the authors from the Web of Science Core Collection (https://www.webofscience.com), using a topic search that excluded the acronym “ILT” due to its ambiguity across multiple fields. As shown in Figure 9, publication activity began to rise notably around 2004, coinciding with the increasing challenges of optical proximity effects (OPEs) as the semiconductor industry scaled to the 90 nm technology node. At that time, conventional model-based OPC—relying on localized edge corrections—struggled to address complex pattern interactions in dense layouts, limiting its correction capability. This limitation spurred interest in ILT as a more holistic resolution enhancement technique. The number of publications peaked in 2010 at approximately 28 articles, and then it basically remains stable, reaching about 23 articles in 2015.

The reasons for this fluctuation can be summarized as follows: First, he anticipated the transition from deep ultraviolet (DUV) to extreme ultraviolet (EUV) lithography—widely expected during the late 2000s and early 2010s—led many to believe that complex RET techniques like ILT would become less critical, as EUV promised reduced optical proximity effects (OPEs). Second, ILT’s high computational complexity and long runtime far exceeded the capabilities of the available hardware and software at the time, making full-chip implementation impractical. Third, ILT-generated masks often feature curvilinear patterns, but commercial mask writing remained dominated by variable-shaped beam (VSB) tools, which are inherently limited to rectilinear (Manhattan-style) geometries due to their aperture-based writing mechanism. While “Manhattanization”—approximating curvilinear features with dense rectilinear segments—was technically feasible, it incurred a significant mask write time and cost, undermining ILT’s practicality. Consequently, ILT research declined, and its industrial adoption was largely confined to localized hotspot correction rather than full-chip implementation. Around 2020, the convergence of several factors revived interest in ILT: the maturation of GPU-accelerated computing, the emergence of deep-learning-based optimization methods, and—critically—the commercial availability of multi-beam mask writing (MBMW) systems capable of efficiently fabricating curvilinear masks. Furthermore, the need for alternative patterning strategies in environments with limited access to cutting-edge lithography infrastructure has further motivated the re-exploration of ILT as a resolution enhancement technique.

**Figure 10:**
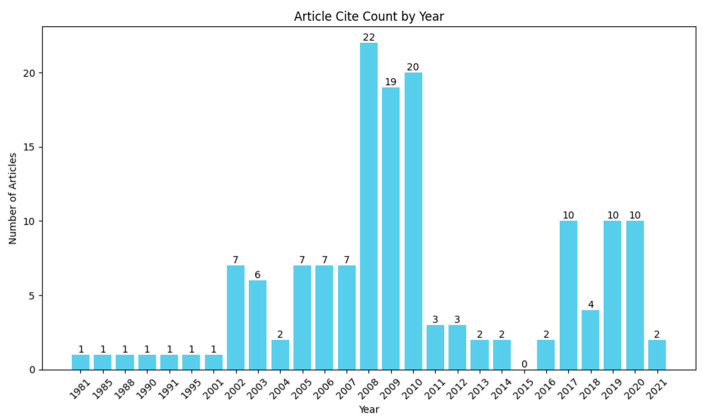
Annual number of publications in the paper “Inverse lithography technology: 30 years from concept to practical, full-chip reality”.

To further corroborate the observed publication trend, the authors analyzed the publication dates of references in the ILT review paper *Inverse lithography technology: 30 years from concept to practical, full-chip reality* published by Linyong (Leo) Pang in 2021 [13]. Given Pang’s extensive involvement in ILT development since the mid-2000s, his 2021 review provides a representative snapshot of the field’s evolution. As review articles typically reflect the state of a field through their citation patterns, the temporal distribution of references serves as an indirect indicator of the historical research activity. Figure 10 shows that the distribution trend of references from different years in this review is consistent with that in Figure 9: the number of citations from 2008 to 2010 was significantly higher than in other periods, followed by a substantial decrease, and then a recovery from 2017 to 2020, suggesting a revival of ILT research in the years leading up to the review’s publication.

**Figure 11:**
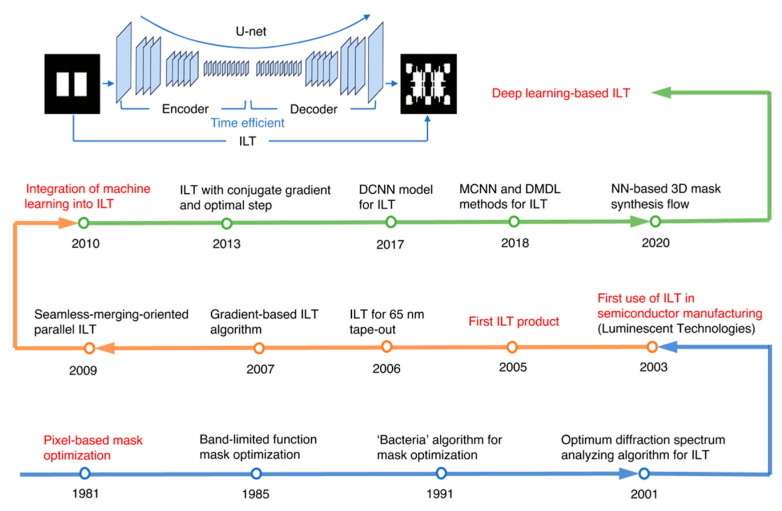
Historical overview of ILT (1981–2020) [43].

Numerous notable milestones have marked the development of ILT. A recent review published in 2025 effectively summarizes these key events and representative advances, as illustrated in Figure 11. For further details, readers are referred to the original article [43].

### 3.3. Key Milestone Events

The important milestone in the development history of ILT not only connects the trajectory of lithography technology’s transition from experience-driven to computation-driven, but also profoundly reshapes the underlying logic of “design and process collaboration” in semiconductor manufacturing.

#### 3.3.1. Initial Concept Proposal

In 1981, Saleh [13] proposed the concept of pixel-based mask optimization, which broke through the traditional forward thinking of “from layout to mask” in photolithography. For the first time, mask design was regarded as an optimization problem that could be deduced through mathematical models, laying the theoretical foundation for ILT. This breakthrough, like equipping photolithography technology with a “calculator”, opened up the possibility of using algorithms to break through physical limits.

#### 3.3.2. Formal Naming and Initial Appearance of ILT Products

In 2003, Luminescent Technologies, Inc. promoted the industrial application of ILT and gave it its official name, marking the transition of this technology from the laboratory to the wafer fab, solving the pain point of the insufficient accuracy of traditional OPC in complex graphics, directly supporting the miniaturization of semiconductor devices to smaller sizes.

Meanwhile, Luminescent initiated the first push to commercialize ILT into real semiconductor manufacturing, with its key algorithm based on level-set methods invented by Osher (a cofounder) and Sethian [18]. Luminescent announced an ILT product at the 2005 Photomask Technology Conference.

#### 3.3.3. Level-Set Method Breakthrough

The level-set method was first introduced to ILT in 2003 by Luminescent: this method represents design, mask, and wafer patterns as level sets of a higher-dimensional function, enabling continuous optimization and the handling of topology changes naturally. This approach significantly reduced the complexity and runtime of ILT optimization while generating curvilinear mask patterns with improved critical dimension (CD) uniformity and larger process margins. Unlike traditional methods that relied on discrete pixel-based representations, the level-set method provided a mathematically elegant solution for solving topology discontinuities during ILT optimization. Its application in ILT marked a shift from rule-based OPC to a more advanced, mathematically rigorous approach, paving the way for full-chip ILT solutions and enhancing the reliability of mask-manufacturing processes [13].

#### 3.3.4. Pixelated Masking Representation

In 2007, Intel [40] launched its own version of ILT and named it “Pixelated Mask Technology”, which reflected the industry giants’ recognition of the value of ILT and promoted its evolution from niche technology to mainstream solutions, especially in the manufacturing of high-resolution and high-complexity logic chips, demonstrating its irreplaceability. Building on the foundational work of academic research and industrial experimentation, Intel’s approach sought to address the computational challenges and practical limitations of earlier ILT methods. The method utilized alternating phase-shift masks (AltPSMs) and a pixel-based design, where the minimum mask feature was a square “pixel” to simplify computation and meet mask-manufacturing rules [13]. Unlike previous methods, such as Luminescent’s level-set approach, Intel’s pixelated method reduced the degrees of freedom to large pixels with only two-phase options (0° and 180°), enabling more efficient optimization. However, the 100 nm pixel size on the mask (25 nm on the wafer) posed challenges due to the strong 3D mask effects, prompting Intel to develop advanced mask 3D modeling techniques. Despite its computational advantages, the method faced limitations in edge-placement accuracy and practical runtime for full-chip designs. Collaborative efforts with D2S and advancements in GPU acceleration, combined with the introduction of multibeam mask writers, eventually overcame these challenges, making full-chip ILT a practical reality. The Intel Pixelated ILT method not only demonstrated the potential of ILT for improving wafer process windows but also paved the way for subsequent innovations in curvilinear ILT and its application to advanced lithography nodes.

#### 3.3.5. Algorithmic Diversification and Computational Advances in ILT

In 2010, Gauda contributed significantly to ILT: they began with GPU-accelerated OPC work [63] and later invented a new approach for solving the ILT problem in the frequency domain [41], unlike Luminescent’s level-set method which operates in the spatial domain. Another team led by Professor Edmund Lam from the University of Hong Kong discussed how various regularization techniques could be used to address two issues: simplifying mask patterns in the inverse lithography process and explicitly incorporating robustness into the design algorithm [64]. They also treated mask design as a machine-learning problem by considering focus variation as a stochastic variable and adopted the stochastic gradient descent approach—a valuable machine-learning tool—for mask design training. Simulations demonstrate that their proposed algorithm is more effective than the previous work in producing robust masks [44]. Luminescent presented results from memory and logic devices at the 32 nm node and below to demonstrate the benefits of level-set-method-based ILT in design rule optimization, SMO, and full-chip correction [65].

#### 3.3.6. Multi-Beam Mask Writing Technology

**Figure 12:**
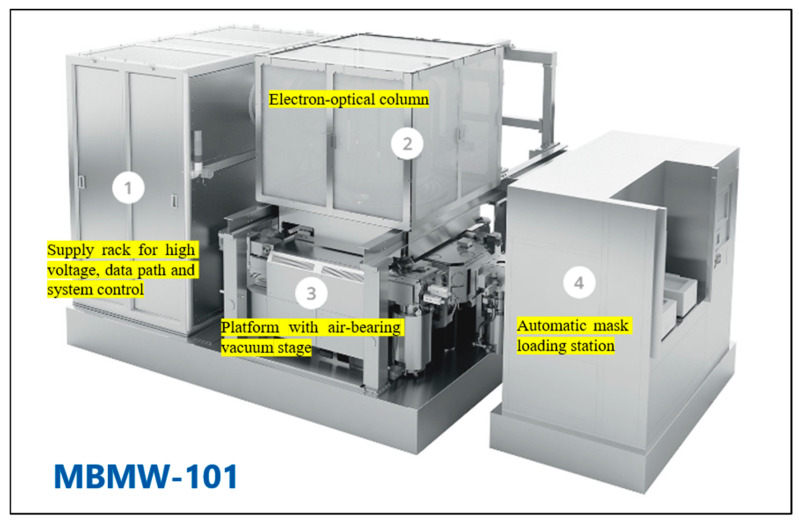
The tool photo of MBMW-101 [68].

Traditionally, variable shaped beam (VSB) mask writers struggled with the time-consuming and costly process of approximating curvilinear shapes using small rectilinear shots, which limited the practical adoption of ILT in production. Recognizing this bottleneck, the photomask industry sought innovative solutions, leading to the emergence of multi-beam mask writer (MBMW) technology [66,67]. Despite the proliferation of multi-beam writing designs in the early 2000s, only the architecture developed by IMS Nanofabrication achieved practical success. In 2013, the company introduced the MBMW Alpha tool, the inaugural electron-beam multi-beam mask writer, which was designed to overcome the technical bottlenecks of the prevailing VSB writers. Introduced in 2016, IMS’s MBMW-101 revolutionized mask writing by employing an array of 262,000 simultaneously operating beams, enabling write times that are nearly independent of pattern complexity. This technology allows for grayscale exposure at each pixel, making it ideal for writing curvilinear ILT patterns with high efficiency and accuracy. By addressing the roadblocks of the write time and pattern fidelity, MBMW technology has accelerated the adoption of ILT, particularly for advanced lithography techniques like EUV, where its shape-agnostic capabilities and superior dose control provide significant advantages. Figure 12 show the MBMW-101 prototype [68].

**Figure 13:**
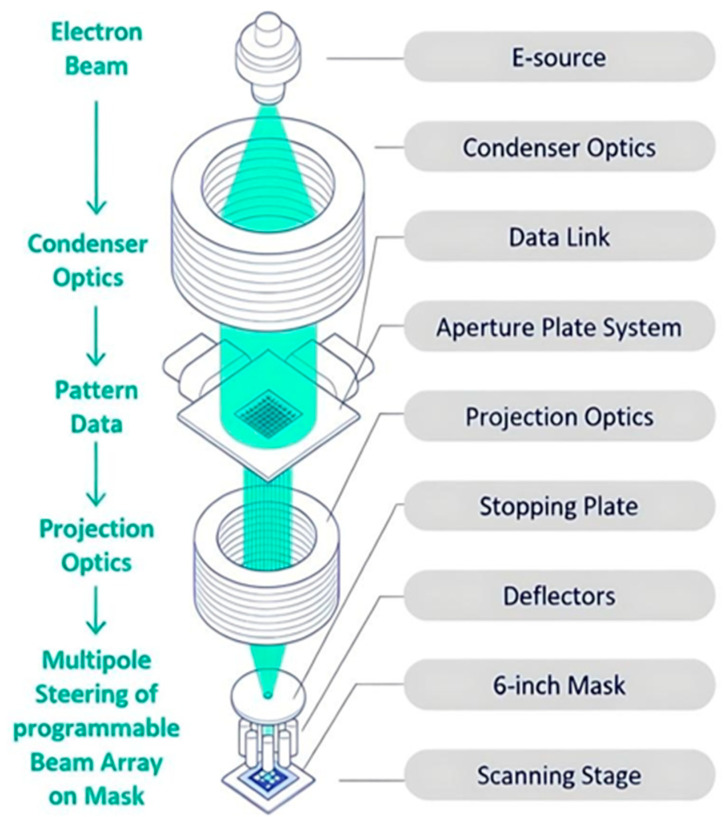
Fundamental concept of multi-beam mask writing [69].

In 2022, NuFlare Technology also launched a MBMW for the industry. Despite being developed by a different manufacturer, this system shares the same fundamental concept as IMS solutions, with each generation of MBMW tools built on an identical electron-optical architecture. Illustrated in Figure 13, the system begins with an electron source that generates a primary beam, which is then expanded and collimated by the condenser optics to produce a uniform, perpendicular electron beam that fully illuminates the aperture plate array. At this stage, the beam is split into several hundred thousand individually addressable microbeams, enabling high-throughput patterning across the mask substrate. The subsequent projection optics demagnify the beam array by a factor of 200× and accelerate the electrons to a final energy of 50 keV before directing them onto the mask. Finally, a multipole deflector steers the entire beam array at high speed, ensuring precise and accurate pattern placement on the mask [69,70].

#### 3.3.7. GPU-Accelerated ILT

**Figure 14:**
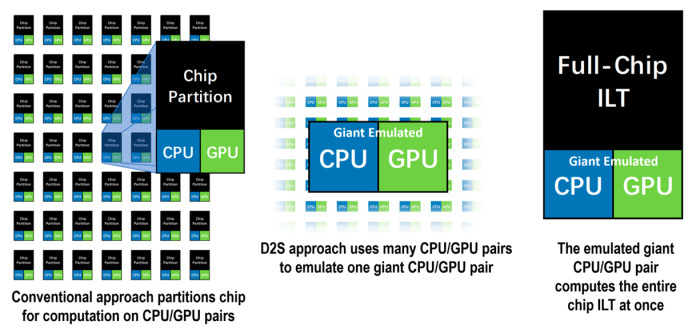
TrueMask ILT GPU–CPU pairs, although comprising many GPU/CPU pairs, has been holistically designed so that it behaves as a single GPU/CPU pair, iterating on the entire chip as a whole, and avoiding stitching errors [42].

In 2019, D2S [42] developed a GPU-accelerated hardware platform, termed the computational design platform (CDP), and crafted the TrueMask ILT software to enable full-chip simultaneous optimization. This approach eliminates the need for time-consuming recursive correction passes traditionally required to address stitching errors. As shown in Figure 14, the D2S CDP has been purpose-built specifically to address simultaneous full-chip optimization. Though it comprises dozens of GPU–CPU pairs, TrueMask ILT—encompassing both the CDP and its software—is engineered to function as a single, massive GPU–CPU pair capable of processing the entire chip’s mask in one go.

#### 3.3.8. Integration of AI/ML

The application of DL to ILT represents a significant advancement in computational lithography, driven by the need to address the challenges of advanced-node lithography. The background of this development lies in the inherent complexity of ILT, which requires solving an iterative optimization problem to determine the optimal mask patterns that produce the desired wafer results. Traditional ILT approaches, while effective, are computationally intensive and time-consuming, particularly for full-chip applications. The advent of DL, inspired by its success in various artificial intelligence applications, has opened new avenues for accelerating and enhancing ILT workflows.

One of the earliest applications of DL to ILT was demonstrated by ASML Brion [52,71,72], where a deep convolutional neural network (DCNN) was used to train an ILT model for generating SRAFs and initializing the ILT engine. This approach significantly reduced the computational burden of ILT by leveraging pre-trained neural networks to guide the optimization process. Subsequently, researchers explored the use of reinforcement learning (RL) to directly generate ILT mask patterns, showcasing the potential of DL to create ILT solutions independently of traditional optimization methods.

The integration of DL into ILT is particularly promising for addressing the challenges of EUV lithography, where the computational demands are significantly higher due to the smaller wavelength and increased pattern density. By leveraging the scalability of GPU-based DL solutions, researchers aim to overcome the runtime limitations of ILT for EUV applications, paving the way for its adoption in next-generation semiconductor manufacturing. Overall, the application of DL to ILT represents a transformative step in computational lithography, offering new opportunities to enhance process windows, improve pattern fidelity, and enable the practical implementation of full-chip curvilinear ILT solutions.

#### 3.3.9. Demonstration of Full-Chip ILT Feasibility

In 2017, Synopsys and SK Hynix [73] presented a significant evaluation of ILT applied to single patterning for DRAM memory devices, particularly focusing on a full-chip random contact layer, demonstrating that adopting ILT enables the process to be completed with only one mask layer. Compared to traditional expensive double-patterning technology (DPT), ILT also preserves the high lithographic production quality while meeting manufacturable OPC/RET production metrics. Key findings include a notable increase in the depth of focus (DoF) by ~20%, with overall CD errors consistently maintained below 5 nm. The ILT approach not only effectively addressed the issues of process window narrowness and poor image profiles prevalent with conventional methods but also allowed for cost reductions by eliminating additional lithographic patterns associated with DPT. This is the first public report on the application of ILT technology to DRAM, solving the problem of full chip production, which enhances the confidence in the continuous investment in ILT research and development.

## 4. Application Status and Challenges

### 4.1. Applications Status of ILT from an Industry Perspective

#### 4.1.1. Hotspot Fixing and SRAF Generation

ILT has evolved significantly over the past three decades, transitioning from an academic concept to a practical solution in advanced semiconductor manufacturing. Initially introduced in the 1980s and further developed in the 1990s, ILT was recognized for its potential to address the challenges of advanced-node lithography, particularly in improving the wafer process window. Despite its theoretical advantages, early implementations faced significant roadblocks, including excessive computational runtimes, the limitations of VSB mask writers in handling curvilinear shapes, and concerns about mask manufacturability.

Due to these reasons, ILT could only be applied initially to critical “hotspots” on the chip [74,75,76]. Later on, ILT has been committed to solving the problem of placing sub-resolution assist features (SRAFs) [52,71,72,77,78,79,80,81]. Recent advancements, however, have transformed ILT into a viable production technology. The introduction of general-purpose graphics-processing unit (GPGPU) computation and MBMW has been instrumental in overcoming these challenges [42]. For instance, in 2019, D2S demonstrated a full-chip ILT solution with practical runtimes (around 48 h) and mask–write times (around 12 h), marking ILT was successfully applied to full-chip production. This breakthrough was further expanded in 2020 with the development of mask-wafer co-optimization (MWCO) techniques, enabling ILT to be applied to masks written by VSB writers while maintaining comparable benefits and practical runtimes [82].

#### 4.1.2. Attempt on Full-Chip

ILT has emerged as a critical computational lithography technique for enhancing the pattern fidelity and process window in sub-10 nm semiconductor manufacturing. Despite its theoretical advantages, widespread high-volume manufacturing (HVM) adoption remains constrained by two primary barriers: a prolonged runtime (often exceeding 100 h for full-chip optimization) and complex curvilinear mask fabrication (requiring multi-beam mask writers like IMS’s MBMW-401 [82]). This paper examines the current deployment status of full-chip ILT across key semiconductor regions, emphasizing the interplay between technological maturity, infrastructure, and regional manufacturing ecosystems.

South Korea, Intel in USA, Taiwan Province of China, and mainland China are at the forefront of ILT’s practical application in full-chip scenarios. For memory chips, ILT has successfully transitioned to full-chip mass production. Leveraging the repetitive array structures of memory devices, manufacturers can adopt modular optimization strategies and parallel computing techniques to effectively control the runtime and reduce development costs. This deployment is particularly widespread at Samsung and SK Hynix, where ILT is utilized to address pattern distortion issues in high-density memory nodes. In the logic chip domain, full-chip application has only been realized by Intel and TSMC, but it is still far from large-scale deployment. The primary barrier lies in the excessive runtime required for full-chip ILT optimization. To address this efficiency issue, Intel has employed a large number of CPUs for parallel computing, while TSMC has adopted GPU acceleration to enhance the computational speed. A notable commonality among these leading enterprises is their in-house mask factories (though SK Hynix’s ownership of an in-house mask manufacturing facility remains unconfirmed), which enables the seamless integration of ILT design and mask fabrication, thereby circumventing runtime limitations and accelerating the technical validation process.

In Mainland China, ILT application remains limited. On one hand, the majority of chip production still caters to the demand for mature-process chips, where the optimization results of traditional OPC can barely meet the manufacturing requirements. Consequently, there are few application scenarios where ILT is deemed indispensable. On the other hand, the lack of supporting equipment infrastructure—such as advanced lithography tools and MBMW—further hinders the widespread adoption of ILT. A key contextual factor is that regions with extensive ILT adoption have entered the EUV era, while EUV access in Mainland China is currently restricted. However, this constraint has, conversely, driven more in-depth research into ILT application in DUV lithography, as the reliance on ILT has increased to address the manufacturing challenges of advanced-node chips under lithography equipment limitations.

#### 4.1.3. ILT in the EUV Era

ILT’s application has also extended to EUV lithography, where its ability to generate curvilinear mask shapes offers significant advantages in improving the process window and addressing challenges such as line-edge roughness. As the semiconductor industry advances toward 3 nm and beyond, ILT is expected to play a critical role in EUV lithography, leveraging GPU-accelerated computing and multi-beam mask-writing technologies to meet the demands of high-volume production.

Fundamentally, there is no essential difference between EUV ILT and DUV ILT in terms of core principles—both achieve mask optimization by inversely deriving from the target wafer pattern to compensate for OPE. The only notable distinction lies in the size of the optimized masks: due to the smaller feature sizes of EUV-processed chips (e.g., 5 nm and smaller nodes), the masks generated by EUV ILT are correspondingly more miniaturized, while strictly complying with the manufacturing constraints of MBMW.

Though theoretically consistent, EUV ILT faces more prominent efficiency challenges in practical applications. The shorter wavelength of EUV light demands finer computational grids to accurately simulate complex optical effects like diffraction and interference. Meanwhile, chip designs for advanced EUV nodes are typically more complex, leading to a substantial surge in computational load and making EUV ILT significantly more time-consuming. Thus, computational acceleration has become an urgent priority for EUV ILT deployment.

Additionally, the resolution improvement of EUV lithography is restricted by its reflective projection structure, which limits the maximum numerical aperture (NA) to 0.55 currently [83]. This NA bottleneck further underscores the necessity of ILT, as it becomes a key supplement to break through the resolution limit of EUV systems and meet the patterning needs of ultra-advanced chip manufacturing.

In summary, ILT has matured into a practical solution for advanced lithography, overcoming historical limitations through technological innovations. Its application is no longer confined to hotspot corrections but now includes full-chip mask generation, curvilinear design, and EUV lithography. As the industry continues to push the boundaries of semiconductor manufacturing, ILT is poised to become an essential tool in achieving higher design densities, improved process windows, and more reliable manufacturing flows.

### 4.2. Challenges and Limitations

ILT has faced several significant challenges and limitations throughout its development and adoption in lithography. These challenges have hindered its widespread use in production environments, despite its promise as a next-generation RET. The primary obstacles can be categorized into computational limitations, mask manufacturing complexities, and practical implementation barriers.

#### 4.2.1. Computational Complexity and Runtime

One of the most significant challenges has been the computational runtime required for ILT. ILT involves solving an inverse problem to determine the optimal mask shapes that will produce the desired wafer patterns. This process is computationally intensive due to the large solution space and the need for iterative optimization. For many years, the runtime for ILT was an order of magnitude longer than that of traditional OPC [84], making it impractical for full-chip applications. This limitation restricted ILT to niche applications, such as correcting critical hotspots on chips, rather than being used for full-chip mask generation. However, advancements in GPU acceleration and purpose-built ILT systems have significantly reduced these runtimes, making full-chip ILT a practical reality in recent years [85].

#### 4.2.2. Mask Manufacturability Constraints

Another major roadblock has been the incompatibility of ILT-generated curvilinear mask shapes with conventional variable-shaped beam (VSB) mask writers. ILT naturally produces curvilinear patterns, which are more optimal for achieving larger wafer process windows. However, VSB mask writers, which dominate mask manufacturing, are designed to write rectilinear shapes. To adapt ILT-generated curvilinear masks for VSB writers, the curvilinear shapes must be “Manhattanized,” or approximated using small rectilinear segments. This process increases the number of VSB shots required to write the mask, leading to impractical write times for full-chip ILT designs. This challenge has been partially addressed with the introduction of MBMW, which can write curvilinear shapes in a shape-agnostic manner, significantly reducing mask write times.

Mask manufacturability has also posed significant concerns. The complex curvilinear shapes generated by ILT are more susceptible to manufacturing variations, such as mask aberrations, which can transfer as systemic errors to the wafer. Additionally, the process of writing curvilinear shapes using VSB writers, which involves creating small rectilinear segments to approximate curves, can introduce inaccuracies in CDs. This has raised worries about the consistency and reliability of ILT-generated masks in production environments. Efforts to mitigate these issues have included optimizing the ILT workflow to reduce the mask complexity, selectively applying SRAFs only where necessary, and leveraging advanced mask inspection and metrology techniques.

In addition, the cost of mask manufacturing is increased due to the need for specialized equipment and processes. The development of new mask manufacturing techniques that can handle the complex patterns generated by ILT while maintaining a high throughput and low cost is an important area of research.

#### 4.2.3. Model Inaccuracy and Calibration Difficulty

The lithography process is a complex multi-physical field coupling process, involving factors such as light–matter interaction, resist chemistry, and thermal effects. Existing models used in ILT often have limitations in fully describing this complex process, leading to a deviation between the designed mask pattern and the actual printed pattern.

For example, the optical imaging models used in ILT may not accurately account for all the optical effects, such as the impact of lens aberrations, partial coherence, and diffraction in different scenarios. The resist models may also not fully capture the complex chemical reactions and physical changes that occur during the exposure and development processes.

In addition, the interaction between different physical fields, such as the coupling between optical and thermal effects, is difficult to model accurately. This can result in the designed mask pattern not producing the expected pattern on the wafer, as the actual process may deviate from the model assumptions. Improving the accuracy of the models by considering more physical effects and their couplings is essential for reducing the deviation between the design and actual results in inverse lithography.

#### 4.2.4. Conservative Market Ecosystem

A conservative market ecosystem poses significant barriers to the widespread adoption and advancement of ILT, particularly for ML-enabled ILT solutions. Firstly, the semiconductor industry’s inherent risk aversion toward emerging technologies has fostered a lack of confidence in ML-based ILT. Foundries, as core players in the supply chain, prioritize process stability and yield assurance over technical innovation; the potential risks associated with unproven technologies—such as unpredictable patterning results or production disruptions—deter them from rashly transitioning from mature traditional OPC to ILT.

Secondly, strict data confidentiality protocols in foundries severely hinder the development of data-driven ML models for ILT. Foundries are obligated to safeguard sensitive customer data, including chip layouts and process parameters, which are critical for training high-performance ML algorithms. This leads to a severe scarcity of high-quality, real-world training data. Most existing ML-related ILT studies still rely on the ICCAD-2013 CAD contest dataset [86], utilizing layout data that is over a decade old. Such outdated data fails to reflect the complexity and process characteristics of advanced technology nodes (e.g., 5 nm and beyond), resulting in ML models that lack practical applicability and generalization capabilities in modern manufacturing scenarios.

Furthermore, the absence of real-chip layouts for cutting-edge nodes exacerbates this challenge. Without access to contemporary layout data that incorporates the latest design rules and process requirements, ML-based ILT struggles to achieve the precision and reliability demanded by industrial applications. The conservative business model of foundries, which prioritizes short-term yield stability and cost control over long-term technical investment, further reinforces the resistance to adopting ILT. This risk-averse mindset, coupled with data scarcity and outdated training resources, collectively limits the technological iteration and industrialization of ILT, impeding its potential to address the patterning challenges of advanced lithography.

## 5. Future Development Directions

### 5.1. Hybrid ILT-OPC-SMO Strategies

A promising future development direction for ILT is the adoption of a hybrid optimization strategy. By combining ILT with OPC and SMO technologies, a balance between patterning precision and computational efficiency can be achieved.

OPC mainly focuses on correcting the mask pattern to compensate for the OPE, while SMO co-designs the source and mask to improve hte lithography performance. Integrating ILT with these technologies can leverage their respective advantages. It can also enhance the process window, making the lithography process more robust to variations. For instance, in some studies, the hybrid source-mask optimization (HSMO) algorithm has been shown to achieve larger process windows, extending the DoF and EL, thus more effectively improving the process robustness of 45 nm immersion lithography systems compared to the mask-only optimization method [87].

To balance the computational cost, mask complexity, and lithographic performance, the semiconductor industry is increasingly adopting hybrid ILT-OPC-SMO strategies that intelligently combine the strengths of ILT, traditional OPC, and SMO. In this approach, SMO is first applied at the full-chip level to define an optimal illumination source tailored to the design’s spatial frequency content. Subsequently, critical regions—such as dense logic gates, SRAM cells, or tight-pitch interconnects—are processed with high-fidelity ILT to maximize process window and pattern fidelity, while non-critical or regular areas are handled by rule-based or model-based OPC for efficiency. This tiered strategy leverages ILT’s superior imaging fidelity in critical regions where high precision is paramount, while containing runtime and mask manufacturability challenges through selective deployment.

### 5.2. Improving Model Accuracy Is Also a Key Factor

The accuracy of ILT is critically dependent on the fidelity of the forward models used to simulate the lithographic process. While ILT shares the same foundational modeling framework as conventional OPC—typically based on the Hopkins theory for partially coherent imaging—it places far greater demands on model precision due to its continuous, gradient-driven optimization over the entire mask domain [1,88]. Unlike OPC, which computes corrections at discrete edge sites, ILT requires accurate sensitivity information at every point in a curvilinear mask, making it highly susceptible to even minor model inaccuracies. To address this, several key modeling strategies have emerged from recent industry practice and expert consensus.

First, mask 3D effects must be explicitly incorporated [89], especially for sub-5 nm nodes and high-NA EUV lithography. Real photomasks possess finite absorber thickness, sidewall angles, and multilayer structures that induce phase shifts, image shifts, and shadowing under oblique illumination—effects ignored by the thin-mask approximation. For ILT-generated curvilinear patterns, which often include fine assist features and dense serpentine shapes, these 3D interactions can significantly distort the printed image. Integrating calibrated Mask 3D compact models or fast electromagnetic field (EMF) solvers into the optical simulation pipeline has thus become essential for predictive accuracy.

Second, there is strong agreement that SRAFs and main printable patterns should be modeled separately. SRAFs are designed not to print; their sole function is to modulate the aerial image of nearby main features. However, their optical scattering behavior and resist response differ fundamentally from those of printable structures due to their small size, isolation, and non-threshold exposure conditions. Using a single unified model for both leads to systematic errors in process window prediction and depth-of-focus balancing. Best practices now recommend dual-path modeling: one path calibrated on SRAF-induced aerial image modulation (often using optical metrics), and another trained on actual printed main features using wafer metrology. This decoupling improves both the model robustness and ILT convergence.

Third, the field is shifting decisively from gauge- or CD-based modeling toward full-contour-based modeling. Traditional workflows rely on CD measurements at predefined gauge points, discarding spatial information about line-edge placement, curvature, and 2D shape fidelity. Yet, ILT optimizes for an entire target contour—not just a few scalar CDs. Calibrating against sparse gauge data creates a fundamental mismatch between optimization objectives and real-world performance. In contrast, contour-based modeling leverages high-resolution SEM images to extract full edge contours, enabling pixel-level or edge-registered loss functions during model fitting. This approach captures subtle effects like line-end shortening, corner rounding, and bridging more faithfully, and provides gradients that better reflect the actual process behavior. Leading foundries now consider contour-based calibration indispensable for advanced-node ILT.

Complementing these strategies, ongoing efforts also focus on improving resist modeling through reaction–diffusion frameworks, incorporating thermal effects during post-exposure bake, and embedding mask manufacturing error models—particularly important for curvilinear masks written by multi-beam writers, where edge placement errors are more complex than in Manhattan geometries. Together, these advances form a more holistic and physically grounded modeling foundation, ensuring that ILT’s theoretical advantages translate into reliable, high-yield patterning on the wafer.

### 5.3. AI-Driven Inverse Design

Since ASML Brion first applied DL to ILT in 2017, the popularity of using ML methods to solve ILT problems has never decreased. The DL has shown great potential in handling complex data and optimization problems. For example, a model-driven convolution neural network (MCNN) can be used to obtain an approximate guess of the ILT solutions [50]. The neural network architecture and initial network parameters are derived from the model-based iterative ILT optimization procedure. The combined approach of using MCNN together with the gradient-based method can improve the speed of ILT optimization algorithms up to an order of magnitude and further improve the imaging performance of coherent optical lithography systems [50].

In addition, other deep-learning-based methods, such as those using generative models [10,54,59] or reinforcement learning (EL) [90], can be explored. Generative models can be used to generate mask patterns directly, while EL can be applied to optimize the lithography process by learning from the interaction between the system and the environment. These AI-driven optimization methods can potentially revolutionize the inverse lithography field by providing more efficient and accurate solutions [44,51,69,71,91,92,93,94,95].

### 5.4. GPU Acceleration as a Core Enabler

**Figure 15:**
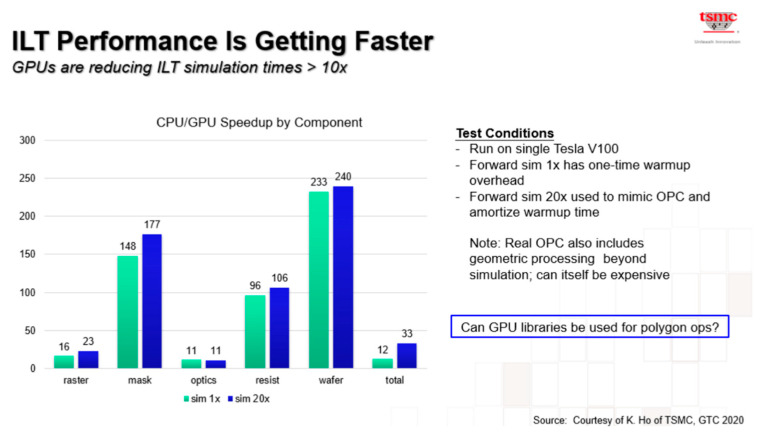
ILT performance is becoming faster with GPUs; GPUs are reducing ILT simulation times > 10× [3].

GPU acceleration is emerging as a core enabler for the practical deployment of ILT. The optimization algorithms in ILT involve massive matrix operations and iterative forward/backward simulations that are inherently parallelizable. By leveraging the high-throughput computing architecture of GPUs, these computations can be dramatically accelerated. In 2023, NVIDIA introduced cuLitho, a GPU-accelerated computational lithography platform, which demonstrated up to 40× speedup in ILT workloads compared to CPU-only implementations [8]. As shown in Figure 15, earlier, TSMC reported that GPU-based acceleration reduced ILT simulation time by over an order of magnitude, enabling a feasible turnaround for critical layers in advanced notes [3]. As chip designs grow more complex and curvilinear masks become standard, the continued co-design of ILT algorithms and GPU hardware will be essential in order to meet high-volume manufacturing requirements [41,63,96,97,98,99].

### 5.5. Maturation of Multi-Beam Mask Writers

Initially, the photomask industry relied on variable-shaped beam (VSB) mask writers, which were limited in their ability to efficiently write curvilinear ILT mask shapes due to their reliance on rectilinear shapes and high shot counts. This limitation significantly hindered the adoption of ILT for full-chip production. The breakthrough came with the introduction of MBMW, which use an array of beams to write patterns in a single shot. These writers are shape-agnostic, meaning they can write any shape, including curvilinear ILT patterns, in a constant write time. The first practical MBMW were introduced around 2016, enabling full-chip ILT to become a reality [66,67]. Companies like IMS, NuFlare played pivotal roles in developing these technologies.

**Figure 16:**
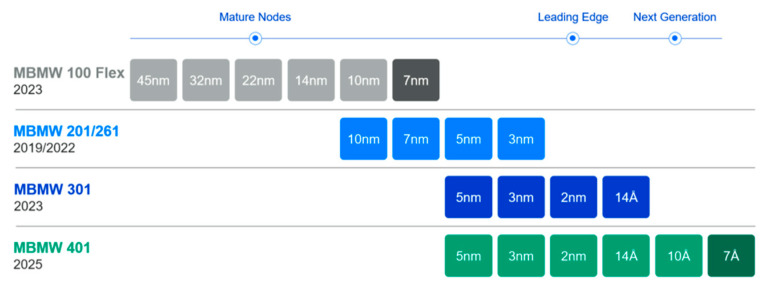
MBMW product family of IMS Nanofabrication; Next-generation multi-beam mask writer MBMW-401 launched in 2025 [69].

IMS Nanofabrication dominates the MBMW equipment market, supplying over 70% of the global capacity for high-volume EUV mask production as of 2023 [100]. This equipment enables the efficient fabrication of curvilinear mask patterns essential for advanced lithography nodes. Since the introduction of its first MBMW alpha version in 2016, IMS/NuFlare has continuously advanced its product line through successive generations. For instance, IMS Nanofabrication’s product series has evolved from the MBMW-101 to the MBMW-201 and MBMW-301, culminating in its latest flagship model, the MBMW-401, which was officially launched in 2025. The MBMW-401 features enhanced beam current and higher data throughput, supports more flexible writing modes, and is capable of meeting mask fabrication requirements across a wide range of technology nodes—covering the 5 nm to 10 Å (1 nm) range for mass production, with R&D capabilities extending even further down to the 7 Å (0.7 nm) node [69]. Figure 16 illustrates the IMS MBMW product family and the corresponding semiconductor nodes each system is designed to support, based on 2025 data.

While MBMWs offer significant advantages, their adoption will depend on cost and practicality. However, as the technology matures and becomes more widely deployed, it is expected to replace VSB mask writers for critical applications, particularly in advanced nodes and EUV lithography. The development of MBMWs has been a transformative force in the photomask industry, overcoming the limitations of VSB mask writers and enabling the practical implementation of ILT. Looking ahead, MBMWs are poised to become the cornerstone of advanced lithography, driving innovation in EUV, curvilinear design, and high-precision manufacturing. Their continued evolution will be crucial for addressing the challenges of next-generation semiconductor manufacturing.

### 5.6. Open-Source Data and Digital Twins

Open-source data emerges as a critical enabler to break through the data scarcity bottleneck plaguing ILT advancement amid a conservative market ecosystem. The current overreliance on outdated datasets like ICCAD-2013 has severely hindered the development of ML-based ILT, as these resources fail to capture the complexity of advanced technology nodes. To address this, the semiconductor industry should draw inspiration from the success of the XiangShan open-source processor project [101]—an initiative that has fostered collaborative innovation by providing accessible open-source hardware designs and verification data to academia and research institutions.

By establishing standardized open-source data repositories for ILT, including representative real-chip layouts, process parameters, and wafer measurement data (e.g., CD-SEM results) from advanced nodes, researchers can obtain high-quality, up-to-date resources for training ML models and validating optimization algorithms. Such open access not only eliminates the data barrier for universities and small research teams but also promotes transparent technical exchange, accelerating the iteration of ILT technologies. Fortunately, several research teams have already adopted similar frameworks to enable the rapid development and evaluation of GPU-accelerated, AI-driven ILT methods [102].

Notably, open-source data sharing should balance confidentiality and accessibility—for instance, anonymizing sensitive customer information while retaining core process characteristics. This approach can reconcile foundries’ data security concerns with the industry’s demand for innovation, paving the way for more practical ML-driven ILT solutions and unlocking the technology’s full potential in advanced lithography.

Digital twins (DTs)—virtual replicas of ILT processes—have been developed using DL to simulate curvilinear mask patterns, enabling the efficient testing and validation of mask equipment and inspection tools [97,103]. By constructing a holistic, real-time synchronized virtual representation of the entire lithographic ecosystem—encompassing light sources, masks, projection optics, photoresist layers, and silicon wafers—DT establishes a closed-loop framework that bridges the gap between ILT’s mathematical modeling and practical lithographic performance.

Linyong Pang et al. [103] first introduced DT technology to semiconductor manufacturing. The purpose is to address the lack of a large amount of data with depth and breadth in the application of DL in the photolithography and photomask industries. They demonstrated the creation of DL-based DT for a mask SEM and for curvilinear ILT. Specifically, the DT is described as a tool developed by D2S using its deep learning kit (DLK) to generate curvilinear ILT mask patterns. These DT results are not directly usable for wafer printing because they may not meet the edge placement error (EPE) and process window requirements. However, their mask pattern shapes are very close to the curvilinear ILT result, making them suitable for testing purposes.

In addition, DT technology may address the key bottlenecks in ILT’s development—slow prototyping cycles, limited adaptability to real-world variability, and siloed design—by leveraging real-time virtual-physical synchronization and multi-physics integration. As semiconductor manufacturing continues to push toward smaller nodes and higher complexity, DT will play an increasingly pivotal role in unlocking ILT’s full potential, accelerating its adoption in full-chip mass production and advancing the frontiers of sub-wavelength lithography.

## 6. Conclusions

Unlike prior surveys that focus primarily on mathematical formulations or idealized simulation results, this review uniquely emphasizes the practical challenges and engineering trade-offs involved in deploying inverse lithography technology (ILT) in real-world semiconductor manufacturing. As a transformative solution in computational lithography, ILT has evolved from a theoretical concept into a key enabler for advanced-node patterning, overcoming the fundamental limitations of traditional lithography at sub-wavelength scales. From an EDA industry perspective, this work systematically synthesizes ILT’s working principles, algorithmic progress, application milestones, and remaining bottlenecks—offering actionable insights for both academic researchers and industrial practitioners.

ILT’s core value lies in its inverse-design paradigm, which grants higher optimization flexibility than traditional OPC. By deriving mask patterns directly from target wafer patterns, ILT effectively breaks resolution barriers, ensures a high pattern fidelity, and expands process windows—advantages that have been validated by its adoption in hotspot fixing, full-chip trials, and EUV-era applications by major foundries (e.g., TSMC and Intel) and memory manufacturers (e.g., Samsung). The four mainstream implementation methods, especially those integrated with machine learning, have further enriched ILT’s technical toolkit in EDA workflows, while interdisciplinary milestones such as level-set algorithm innovation, pixelated mask technology, multi-beam mask writers, and GPU acceleration have collectively pushed ILT toward industrialization.

Despite these advancements, ILT still faces unresolved bottlenecks that hinder its large-scale mass production. To guide future research, the three most important open challenges are summarized in the following outlook box:

**Outlook Box: Key Open Challenges for ILT Research**

- Computational acceleration: Full-chip ILT optimization remains plagued by an excessive runtime, demanding more efficient algorithms (e.g., hybrid ILT-OPC-SMO) and hardware acceleration (e.g., GPU-centric computing) to meet EDA tool efficiency requirements.
- Mask manufacturability: The complex curvilinear masks generated by ILT pose great challenges to fabrication; advancing multi-beam mask writing technology and optimizing mask design for manufacturability are critical for industrial adoption.
- Accurate multi-physics modeling: The existing models struggle with inaccuracies under real-world process variability (e.g., EUV reflective projection effects and lithography-etch interactions), requiring more precise multi-physics models calibrated with practical industrial data.

Looking forward, ILT’s advancement will depend on multi-technology synergy in the EDA field. AI-driven optimization and GPU-centric computing will address computational bottlenecks; hybrid strategies combining ILT with SMO will enhance holistic performance; and integration with digital twins will bridge the gap between virtual simulation and physical manufacturing. With continuous innovation to overcome the current limitations, ILT will serve as a cornerstone of advanced semiconductor manufacturing EDA tools, playing an increasingly pivotal role in supporting the miniaturization of chip nodes and driving the industry toward higher precision and efficiency.

## Figures and Tables

**Table 1:** Concise comparison of mainstream ILT implementation methods.

ILT Methods	Computational Efficiency	Mask Complexity (Manufacturability)	Applicable Scenarios
Level-Set Method	Medium; stable convergence	Medium; good manufacturability	Medium–small critical patterns, DUV extension nodes
Pixelated ILT	Low; high hardware dependency	High; needs post-processing	Advanced logic full-chip, ultra-strict EPE repair
Frequency-Domain Curvilinear ILT	High; fast for periodic patterns	Low–medium; excellent manufacturability	Memory cell arrays, large-area regular patterns
ML-Assisted ILT	High (inference); low (training)	Medium; controllable with constraints	Mass production hotspot repair, repetitive patterns

## Data Availability

No new data were created or analyzed in this study. Data sharing is therefore not applicable to this article.

## Abbreviations

The following abbreviations are used in this manuscript:

ILT	Inverse Lithography Technology
IC	integrated circuit
OPE	optical proximity effect
OPC	optical proximity correction
RB-OPC	rule-based OPC
MB-OPC	model-based OPC
SRAF	sub-resolution assist feature
EPE	edge placement error
RET	resolution enhancement technique
PB-OPC	pixel-based OPC
CF	cost function
DMDL	dual-channel model-driven deep learning
HDP	hybrid dynamic priority
SMO	source mask optimization
S-Litho	Sentaurus Lithography
POCS	projection onto convex sets
SGD	stochastic gradient descent
Alt-PSMs	alternating phase-shift masks
MRC	mask-rule checking
MCNNs	Model-driven Convolutional Neural Networks
DL	deep learning
CNNs	convolutional neural networks
VAE	variational autoencoder
GAN	generative adversarial network
EUVL	extreme ultraviolet lithography
EBL	electron beam lithography
NIL	nanoimprint lithography
J-FIL	Jet and Flash Imprint Lithography
DUV	deep ultraviolet
VSB	variable shaped beam
SMO	source-mask optimization
MBMW	multi-beam mask writers
DCCS	dense concentric circle sampling
MWCO	Mask-Wafer Co-Optimization
CDP	computational design platform
RL	reinforcement learning
DPT	Double Patterning Technology
DoF	depth of focus
CD	critical dimension
EL	exposure latitude
GPGPU	general-purpose graphics-processing unit
EUV	extreme ultraviolet
HSMO	hybrid source mask optimization
CBCT	cone-beam computed tomography
DT	digital twins
SEM	scanning electron microscope
DPK	deep-learning kit
